# Overview of the winter wave of 2009 pandemic influenza A(H1N1)v in Vojvodina, Serbia

**DOI:** 10.3325/cmj.2011.52.141

**Published:** 2011-04

**Authors:** Vladimir Petrović, Zorica Šeguljev, Gorana Ćosić, Mioljub Ristić, Jasminka Nedeljković, Nataša Dragnić, Snežana Ukropina

**Affiliations:** 1 Autonomous Province of Vojvodina, Provincial Secretariat for Health, Novi Sad, Serbia; 2 Institute of Public Health of Vojvodina, Center for Disease Control and Prevention, Novi Sad, Serbia; 3 Institute of Vaccines, Virology and Sera, Torlak, Belgrade, Serbia; 4 Institute of Public Health of Vojvodina, Center for Biostatistics and Informatics, Novi Sad, Serbia; 5 Institute of Public Health of Vojvodina, Center for Health Promotion, Novi Sad, Serbia

## Abstract

**Aim:**

To analyze the epidemiological data for pandemic influenza A(H1N1)v in the Autonomous Province of Vojvodina, Serbia, during the season of 2009/2010 and to assess whether including severe acute respiratory illness (SARI) hospitalization data to the surveillance system gives a more complete picture of the impact of influenza during the pandemic.

**Methods:**

From September 2009 to September 2010, the Institute of Public Health of Vojvodina conducted sentinel surveillance of influenza-like illnesses and acute respiratory infections in all hospitalized patients with SARI and virological surveillance of population of Vojvodina according to the European Centers for Disease Control technical document.

**Results:**

The pandemic influenza outbreak in the province started in October 2009 (week 44) in students who had returned from a school-organized trip to Prague, Bratislava, and Vienna. The highest incidence rate was 1090 per 100 000 inhabitants, found in the week 50. The most affected age group were children 5-14 years old. A total of 1591 patients with severe illness were admitted to regional hospitals, with a case fatality rate of 2%, representing a hospitalization rate of 78.3 per 100 000 inhabitants and a mortality rate of 1.6 per 100 000. Most frequently hospitalized were 15-19 years old patients, male patients, and patients with pneumonia (*P* < 0.001). The highest case fatality rate was found among patients with acute respiratory distress syndrome (*P* < 0.001). Nasal/throat swabs were obtained for polymerase chain reaction test from 315 hospitalized patients and 20 non-hospitalized patients, and 145 (46%) and 15 (75%) specimens, respectively, tested positive on A(H1N1)v.

**Conclusion:**

Sentinel influenza-like illness and SARI surveillance, both followed with virological surveillance, seem to be the optimal method to monitor the full scope of the influenza pandemic (from mild to severe influenza) in Vojvodina.

In April 2009, a new influenza virus of swine origin was identified in Mexico and the United States. Since then, widespread community transmission of the virus has been confirmed on all continents, and the World Health Organization (WHO) announced a global influenza pandemic (1).

In May 2009, Serbia implemented enhanced national surveillance of influenza A(H1N1)v according to the WHO pandemic plans. The national guidelines on requirements and procedures for reporting individual cases of influenza A(H1N1)v using WHO case definition were created on April 27, 2009. Every individual suspicious case was daily reported by the district Institute of Public Health to the national Institute of Public Health. The results of laboratory testing were daily reported by The National Virology Reference Laboratory. Confirmed and possible cases were categorized as travel-related or domestic (2,3).

The first case of pandemic influenza A(H1N1)v in Vojvodina was registered on June 24, 2009. Till August 17, 123 pandemic influenza A(H1N1)v cases were registered and 61/113 (54%) were confirmed by reverse transcription polymerase chain reaction in the reference laboratory. The majority of cases (73%) were imported during the EXIT international music festival. These cases were registered among domestic population epidemiologically linked to the music festival. Some of the cases did not meet the definition of suspicious cases, which means that there were unrecognized cases and that further transmission of pandemic influenza A(H1N1)v was inevitable (3).

At the end of October 2009, the mandatory outbreak investigation of acute respiratory illness detected new cases of pandemic influenza A(H1N1)v among students who had returned from school-organized trips to Prague, Bratislava, and Vienna. This was considered the beginning of the outbreak in the season of 2009/10.

This article focuses on the winter wave of pandemic influenza A(H1N1)v in the Autonomous Province of Vojvodina, northern Serbia, with 2 million inhabitants. We present the outpatient (community) data provided by the sentinel physicians and inpatient data provided by hospitals.

## Methods

Pandemic influenza A(H1N1)v surveillance in Vojvodina, Serbia, during the fall and winter 2009/10 was conducted according to European Centers for Disease Control technical document issued in September 2009 (4). Data for this observational study were obtained from the sentinel surveillance of influenza-like illness (ILI) and acute respiratory infections (ARI), surveillance of all hospitalized severe acute respiratory illness (SARI) cases, and virological surveillance among the population of Vojvodina.

### Sentinel surveillance of ILI and ARI

Sentinel surveillance in Vojvodina first started in the season of 2004/05 and closely relied on the Slovenian surveillance program (5). While the sentinel surveillance is usually conducted from the first week in November to the end of April, during the season 2009/10 it was conducted from September 2009 to September 2010. The involved 103 sentinel physicians were general practitioners and pediatricians from 19 health centers covering municipalities with more than 30 000 inhabitants. The sentinel physicians were selected according to the number of contracts with the inhabitants who chose them as personal physicians. Of the total population of Vojvodina of 2 031 992 according to 2002 census, 102 723 people or 5.1% were monitored. Sentinel physicians tracked cases of ILI and ARI in the age groups 0-4, 5-14, 15-64, and older than 65 years. The number of inhabitants under surveillance (102,723) was used as a denominator for calculation of the weekly incidence of ILI and ARI and for the cumulative incidence for the whole observed period. However, if a sentinel physician did not send a report in a week, the denominator in that week was decreased for the number of inhabitants monitored by the physician without monitoring the registered cases.

Sentinel physicians sent weekly reports (every Monday for the previous week) on the number of cases in each age group to the sentinel surveillance coordinators in district Institutes of Public Health and to the network sentinel surveillance coordinator in the Institute of Public Health of Vojvodina. Weekly incidence rates and age-specific incidence rates were calculated. Geographical spread of the virus and the intensity of ILI and ARI were monitored and compared with the epidemic threshold of incidence of 246.3/100 000 , which was established in the previous 5 sentinel seasons. Weekly incidence of 246.3/100 000 was considered as an influenza activity of medium intensity and as the beginning of the epidemic. Influenza intensity level and virus geographical spread were further determined according to the European Centers for Disease Control technical document (4). The Institute of Public Health of Vojvodina sent weekly feedback report to coordinators in district Institutes of Public Health, to each sentinel physician, and to the national Institute of Public Health. Every weekly report was published at the Institute of Public Health of Vojvodina Web site.

### ILI and ARI case definition

ILI is defined as a sudden onset of high fever (above 38°C), followed by myalgia and arthralgia, dry cough, and other symptoms of upper respiratory tract infections (6). ARI was diagnosed in every case in whom the physician established the following diagnoses (according to International Classification of the Disease, 10th revision): B34, J00, J01.0-J01.9, J02, J04.0-J04.2, J05, J05.0, J06, J06.0, J06.8, J06.9, J12, J12.0, J12.1, J12.2, J12.8, J12.9, J20, J20.3-J20.9, J21, J21.0, J21.8, J21.9, J22.

### SARI surveillance

SARI surveillance started with the initial SARI case registered (June 2009) and followed up each SARI case till September 2010. According to the national program, every SARI case was hospitalized. Hospital coordinators for SARI surveillance from all 15 acute care hospitals in the province sent daily reports on every hospitalized SARI case to the district Institute of Public Health, such as the date of onset of illness, risk factors (age, asthma, chronic obstructive pulmonary disease, chronic cardiovascular disease without hypertension, obesity regardless of level of obesity, pregnancy, diabetes mellitus, malignancies, chronic neurological disorders, malnutrition, and HIV infection), the length of hospitalization, results of laboratory virology tests, and confirmed influenza-related deaths. Individual reports on hospitalized cases with SARI were registered in a computer database in the district Institutes of Public Health and in the Institute of Public Health of Vojvodina.

The following SARI case definition was used (7): *“*A person with ILI and difficulty breathing, demanding hospitalization and/or treatment in intensive care unit with no other etiological agent confirmed was considered to have SARI*.”*

### Virological surveillance

Virological surveillance was conducted in collaboration with sentinel physicians, hospitals, Institutes of Public Health, and the national reference influenza laboratory of the Institute of Virology, Vaccines, and Sera, Torlak, Belgrade. Nasal and throat swabs from sentinel surveillance cases (outpatients) were taken when the definition of ILI was met, at most 2 samples per week from the monitored population in the province. Non-sentinel nasal/throat swabs were taken from patients with SARI (inpatients) in each case of acute respiratory distress syndrome (ARDS) and non-ARDS patients with pneumonia. Both nasal and throat swabs from one patient were placed in the same vial with transport medium and kept on -20°C. Transport of samples was organized by district Institutes of Public Health to the national reference laboratory on a daily basis. Real time reverse transcription polymerase chain reaction was used with Centers for Disease Control (Atlanta, GA, USA) primers and probes using SuperScript III RT/Platinum Taq Mix Stratagene Mx 3005P machine to test the presence of influenza A(H1N1) virus (8). Results were obtained within a few hours and reported daily according to surveillance hierarchy to the national Institute of Public Health, Institute of Public Health of Vojvodina, district Institutes of Public Health, the sentinel/ hospital physician, and finally to the patient.

### Public health measures

One of the public health measures undertaken to decrease viral activity and pressure on hospitals was school closure. The measure was undertaken after extra classes had been planned for the rest of the semester to enable the educational authorities to finish the semester.

Vaccination was also performed, according to the national guidelines made with the recommendation of the WHO (9), as well as methods of social marketing and health education considering segmented population groups (preschool children, younger and older school children, adults, and elderly people).

### Statistical analysis

The weekly incidence of ILI and ARI and age-specific incidences for monitored age groups were measured per 100 000 population. The numerator was the number of the clinical cases of ILI and ARI in the total population and in the age groups. The denominator was the whole population monitored in a week and monitored age groups when age-specific incidences were calculated.

The numerator for the cumulative incidence was all ILI cases in the observed period and the denominator was the monitored population of 102 723 inhabitants.

The rate of hospitalization was the number of hospitalized patients with SARI divided by the total population of districts and the province, specified by sex and age per 100 000 population, according to 2002 census.

The mortality rate was the number of deaths divided by the total population of districts and the province specified by sex and age per 100 000 population, according to the census in 2002.

Case fatality rate (CFR) in hospitalized SARI patients was the proportion of deaths of confirmed cases and the number of hospitalized patients by district, sex, and age. The obtained data were statistically processed using SPSS, version 14.0 (SPSS Inc., Chicago, IL, USA). The results are presented as frequency, percent, and mean ± standard deviation with 95% confidence interval of mean. Difference among incidences was tested by χ^2^ test and difference between sample means by *t* test and ANOVA. In post-hoc analysis, we used least significant difference test. Two-tailed *P* values <0.05 were considered to be significant.

## Results

### Spread of influenza A(H1N1)v

**Figure 1:**
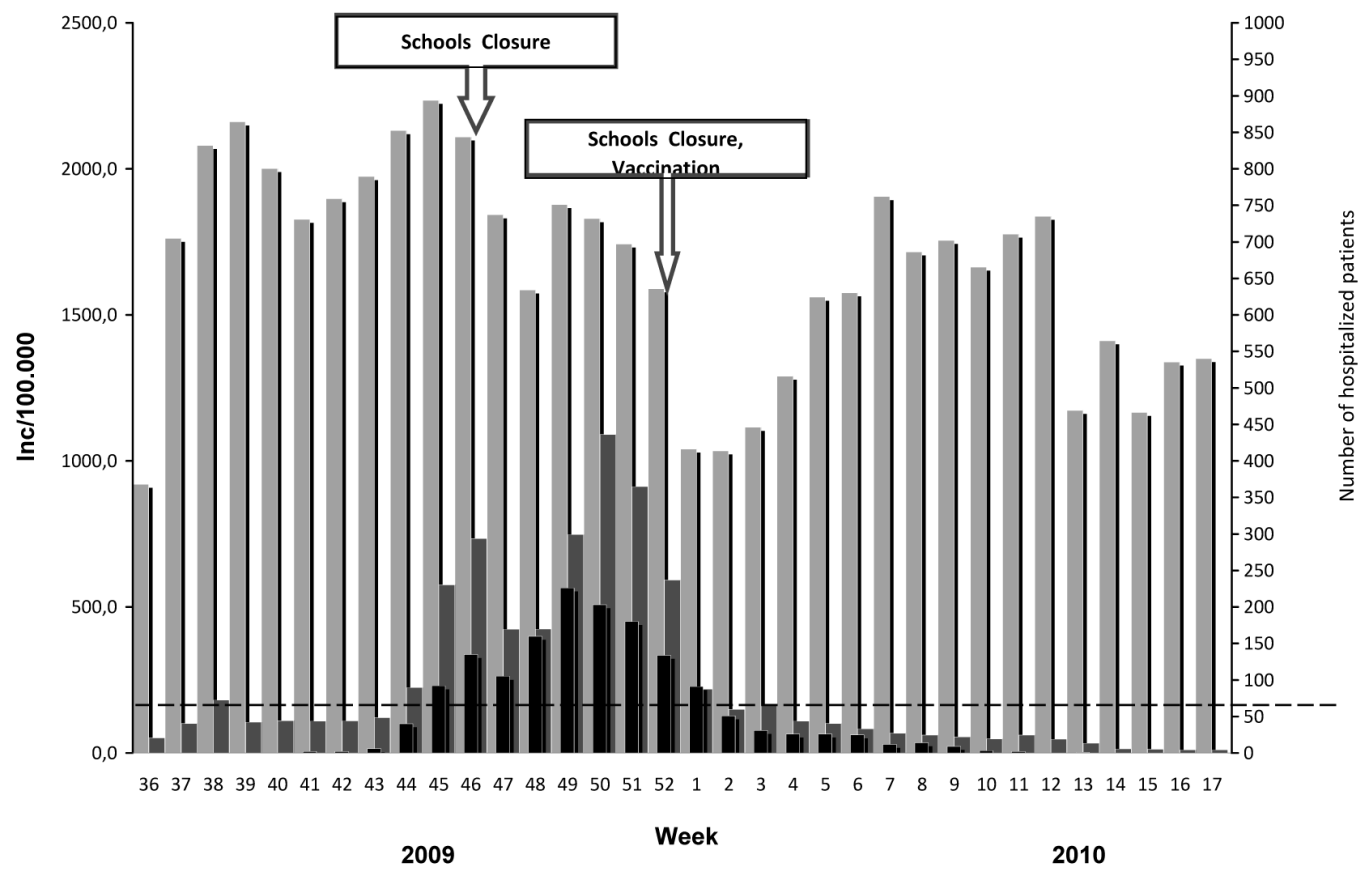
Weekly Incidence rates of influenza-like illness and acute respiratory infections in Vojvodina and the number of hospitalized patients, 2009/10 influenza season. Light gray bars – acute respiratory infections; dark gray bars – influenza-like illness; closed bars – number of hospitalized patients; interrupted line – epidemic threshold.

From October 27 to November 2 (week 44 in 2009), the sentinel surveillance system recorded an increase in the incidence of ILI in Vojvodina, reaching 221/100 000 inhabitants, close to the epidemic threshold (Figure 1).

**Figure 2:**
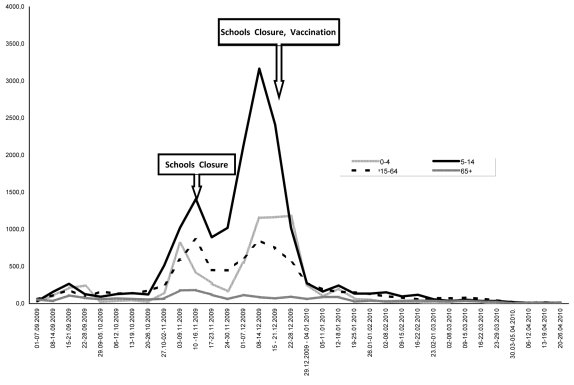
Age-specific influenza-like illness incidence in Vojvodina during 2009/10 influenza season, based on sentinel surveillance. Light gray line – 0-4 age group, full line – 5-14 age group, dashed line – 15-64 age group, dark gray line – ≥65 years.

The epidemic lasted from the week 45 till week 52 of 2009, when the weekly incidence of ILI was above the epidemic threshold. The highest incidence rate was registered in the week 50 of 2009, reaching 1090/100 000. The cumulative incidence rate during the outbreak was 6696.5/100 000. Weekly age specific incidence rates were highest in the age group 5-14 years (*P* < 0.001) (Figure 2).

**Figure 3:**
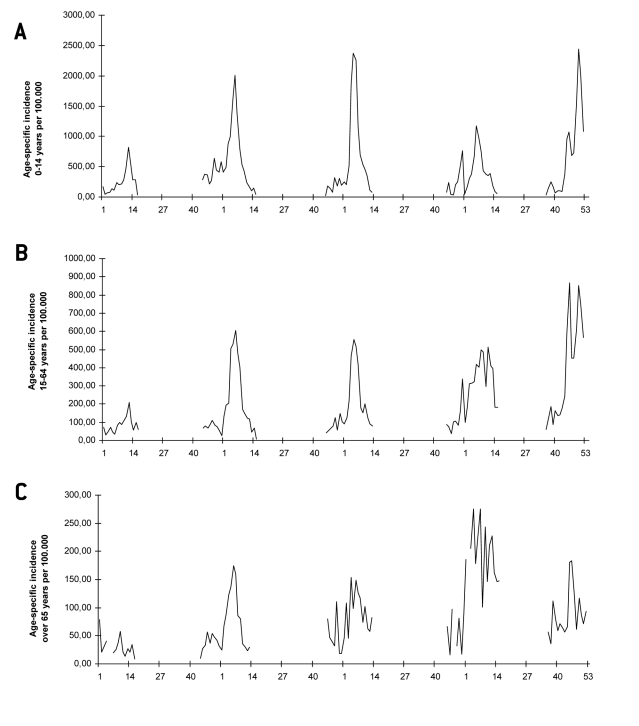
Age-specific incidence of influenza-like illness per 100 000 in Vojvodina during 5 influenza seasons based on sentinel surveillance in age groups: (**A**) 0-14 years; (**B**) 15-64 years; (**C**) over 65 years.

During the last five seasons, influenza epidemics were mild. The highest weekly age-specific incidence of ILI in children 0-14 years old and adults 15-64 years old was registered in 2009/10 pandemic influenza season, while in adults older than 65 years it was registered in 2008/09 influenza season (Figure 3). Cumulative age-specific incidence in adults aged 15-64 years was higher in 2009/10 influenza season than in previous seasons (Table 1).

**Table 1:** Cumulative age specific incidence of influenza-like illness in Vojvodina during 5 influenza seasons, based on sentinel surveillance

Season	Age groups (years)		
	0-14	15-64	65+
2005/06	3996.0	1346.2	436.9
2006/07	1384.2	4393.5	140.3
2007/08	12 292.4	3856.0	1568.2
2008/09	8492.7	6017.6	5126.3
2009/10	11 842.4	6449.6	1515.8

### Hospitalized cases and deaths due to SARI

During the season 2009/10, 1591 patients with SARI were admitted to 15 hospitals with a rate of hospitalization of 78.3/100 000. The ratio of hospitalization in districts was 1:11.6 and ranged from 14.0 to 162.5 per 100 000 (*P* < 0.001).

The number of deaths among cases was 32, with an overall mortality rate of 1.6 per 100 000 and CFR in hospitalized cases of 2%. All deaths were laboratory confirmed. CFRs were significantly different across districts but mortality rates were not (Table 2).

**Table 2:** Hospitalization rate, mortality rate in hospitalized cases, and case fatality rate (CFR) in Vojvodina during 2009/10 influenza A(H1N1) season by district, age, and sex

Demographic variable	No. of monitored population	Rate of hospitalization*	*P*^†^	No. of deaths (mortality rate)*	*P*^†^	CFR in hospitalized cases (%)	*P*^†^
District:							
South Bačka	593 666	6.5	<0.001	10 (1.7)	0.98	2.5	<0.001
South Banat	313 937	162.5		5 (1.6)		1.0	
Central Banat	208 456	154.9		3 (1.4)		0.9	
West Bačka	214 011	29.9		2 (0.9)		3.1	
North Bačka	200 140	14.0		4 (2.0)		14.3	
North Banat	165 881	91.6		2 (1.2)		1.3	
Srem	335 901	35.4		6 (1.8)		5.0	
total	2 031 992	78.3		32 (1.6)		2.0	
Sex:							
male	984 942	88.3	<0.001	20 (2.0)	0.11	2.3	0.37
female	1 047 050	68.9		12 (1.1)		1.7	
total	2 031 992	78.3		32 (1.6)		2.0	
Age (years):							
0-4	92 575	87.5	<0.001	0 (0.0)	0.04	0.0	0.01
5-14	229 630	112.3		1 (0.4)		0.4	
15-19	137 777	120.5		0 (0.0)		0.0	
20-64	1 248 254	77.1		28 (2.2)		2.9	
65+	323 756	37.4		3 (0.9)		2.5	
total	2 031 992	78.3		32 (1.6)		2.0	

*Per 100 000 population.

†The values for demographic variables were compared using χ^2^ test.

Male/female sex ratio of hospitalized patients was 1.2:1. Hospitalization rate of men was higher (*P* < 0.001), but mortality rates were not significantly different between the sexes.

The highest age-specific hospitalization rate (120.5/100 000 ) was found in the 15-19 years age group (*P* < 0.001), while the highest age-specific mortality rate (2.2/100 000 ) was found in the 20-64 years age group (*P* = 0.04). There was a significant difference in CFR in hospitalized cases between different age groups (Table 3) (*P* = 0.01).

**Table 3:** Structure of hospitalized cases and deaths and case fatality rate (CFR) in Vojvodina during 2009/10 influenza A(H1N1) season by observed risk factors and clinical forms

Clinical form or presence of risk factors	No. (%) of hospitalized cases	P*	No. (%) of deaths	P*	CFR of hospitalized cases (%)	P*
Clinical form:						
acute respiratory distress syndrome	62 (3.9)	<0.001	31 (96.9)	<0.001	50.0	<0.001
pneumonia	942 (59.2)		1 (3.1)		0.1	
acute febrile illness	587 (36.9)		0 (0.0)		0.0	
total	1591 (100.0)		32 (100.0)		2.0	
Risk factor:^†^						
any	536 (33.7)	<0.001	24 (75.0)	<0.001	4.5	<0.001
no	1055 (66.3)		8 (25.0)		0.8	
total	1591 (100.0)		32 (100.0)		2.0	

*The values were compared using χ^2^ test.

†Age, asthma, chronic obstructive pulmonary disease, chronic cardiovascular disease without hypertension, obesity regardless of level of obesity, pregnancy, diabetes mellitus, malignancies, chronic neurological disorders, malnutrition, and HIV infection.

ARDS was registered in 3.9% and pneumonia in 59.2% of hospitalized patients with SARI. There was a significant difference in the number of deaths (*P* < 0.001) and CFR of hospitalized cases between different clinical forms of illness, such as ARDS, pneumonia, and SARI without pneumonia (*P* < 0.001). There were no registered deaths among patients with SARI without ARDS or pneumonia.

The majority of hospitalized cases (66.3%) had no risk factors. Deaths due to influenza A(H1N1)v were more frequently registered in patients with one or more risk factors than in patients without risk factors (75% vs 25%, *P* < 0.001) (Table 3).

There were no significant differences in the average duration of illness before hospitalization and the length of hospitalization in patients with SARI by sex (Table 4). Post-hoc analysis showed that there was a significant difference in the average duration of illness prior to hospitalization between the age groups 5-14 years and 20-64 years (*P* < 0.001) and also between the age groups older than 65 and 5-14 years (*P* = 0.035). The difference in the average length of hospitalization was significant between all age groups, except between the age groups 0-4 and 15-19 years.

**Table 4:** Duration of illness for hospitalized cases in Vojvodina during 2009/10 influenza A(H1N1) season by sex, age groups, and clinical form*

Demographic variable or clinical form	Mean (±SD) duration of illness prior to hospitalization*	*P*^†^	Mean (±SD) length of hospitalization*	*P*^†^	Mean (±SD) duration of illness in hospitalized cases	*P*^†^
Sex:						
male	3.3 ± 3.0	0.96	8.1 ± 6.4	0.41	11.4 ± 7.2	0.42
female	3.3 ± 3.0		8.3 ± 6.6		11.7 ± 7.4	
total (95% confidence interval)	3.3 ± 3.0 (3.2-3.5)		8.2 ± 6.5 (7.9-8.5)		11.5 ± 7.3 (11.2-11.9)	
Age:						
0-4	3.1 ± 3.5	<0.001	7.3 ± 12.2	<0.001	10.3 ± 12.2	<0.001
5-14	2.7 ± 3.3		5.4 ± 2.5		8.1 ± 4.3	
15-19	3.1 ± 3.9		6.7 ± 4.3		9.9 ± 5.9	
20-64	3.5 ± 2.8		8.8 ± 6.3		12.4 ± 7.0	
65+	3.4 ± 2.5		11.7 ± 7.9		15.4 ± 8.6	
total	3.3 ± 3.0		8.2 ± 6.5		11.5 ± 6.4	
Clinical form:						
acute respiratory distress syndrome	4.5 ± 2.9	<0.001	16.4 ± 14.7	<0.001	21.2 ± 15.2	<0.001
pneumonia	3.8 ± 3.3		8.9 ± 5.0		12.6 ± 6.0	
acute febrile illness	2.5 ± 2.2		6.2 ± 6.3		8.7 ± 6.6	
total	3.3 ± 3.0		8.2 ± 6.5		11.5 ± 7.3	

*SD – standard deviation.

†The values were compared using *t* test (sex) and ANOVA (age, clinical form).

Post-hoc analysis showed that patients with SARI who developed ARDS and pneumonia had significantly delayed admission at the hospital and longer hospitalization than patients with SARI with only acute febrile illness (*P* < 0.001).

### Laboratory results

A total of 315 swabs were taken from hospitalized patients with SARI (non-sentinel) and 20 swabs from sentinel patients, and 145 (46.0%) and 15 (75%) patients, respectively, had a positive polymerase chain reaction result. The presence of the virus was confirmed in patients from 31 out of 45 municipalities, covering 86.1% of the population.

### Public health control measures

Public health control measures implemented after the outbreak were enhanced support for vaccination; distribution of information leaflets as the main control measures for the preschool and school facilities; distribution of posters in the public transport facilities; appearance of experts in the prime news of the public media; broadcasting videos and jingles on behavior in public indoor spaces, the use and disposal of tissues, and the position of hands when sneezing and coughing; and issuing warnings about the symptoms of influenza and procedures in case of disease recognition.

The Institute of Public Health of Vojvodina Web site weekly presented updated epidemiological reports on the number of cases and issued warnings about a possible further increase.

Schools we closed on two occasions (Figure 1), for one and three weeks, respectively. After the school closure, the epidemic curve showed a decrease in the weekly incidence rate of ILI and the number of hospitalized cases.

**Figure 4:**
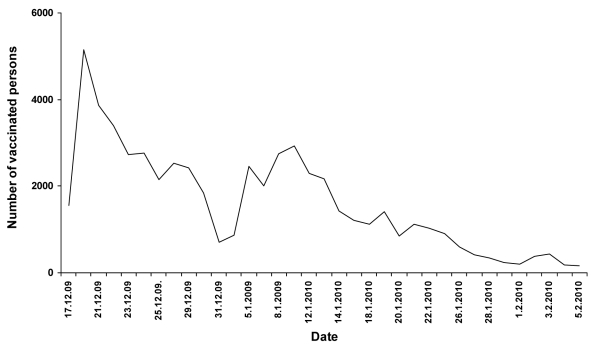
Course of immunization in Autonomous Province of Vojvodina during the influenza season of 2009/10. The line represents the number of vaccinated persons.

Immunization started in the week 51 of 2009, with 2.8% coverage (Figure 4).

## Discussion

Our study found that school closure reduced the intensity of clinical activity and pressure on hospitals. The fatal outcome was more common among patients with one or more risk factors and the outcome and severity of the disease depended on the time elapsed from the onset of illness to the hospital admission and therapy.

In our country, the comparison of the 2009/2010 influenza season with previous seasons cannot be based solely on hospital-based surveillance data, as there are no reliable records on hospital admissions and deaths related to laboratory-confirmed influenza. Sentinel surveillance is a good method to detect the beginning and the severity of the epidemic, similar to the findings of other authors (10). The fact that the 2009/2010 epidemic season began two to three months earlier than previous seasons could be explained by the introduction of a new surveillance method. Using the same case definitions, it was possible to compare the current situation with the previous period (11,12).

The weekly incidence of ILI above the epidemic threshold in the majority of districts and the incidence rate of 1090.0/100 000 inhabitants in the week 50 of 2009 indicates a widespread geographical activity of the virus and a very high intensity of clinical activity. Cumulative incidence was used to estimate 136 000 clinically manifested cases in Vojvodina.

A limitation of this study is that we were not able to confirm a larger number of cases because only one laboratory in Serbia was designated and equipped for the confirmation of pandemic influenza virus during 2009/10 influenza season and we had to rely on clinical case definition of influenza-like illness.

In most reports, the increased incidence among young people could be explained by the fact that more young people are tested, for example during outbreaks of influenza in schools (13-15). We believe that in our province the outbreak of pandemic influenza started in students who had returned from school-organized trips abroad in October 2009. Large numbers of reservoirs were imported in a short period, enabling the outbreak occurrence rather than silent transmission that happened in the population during the summer of 2009, after the outbreak among music festival participants (3). Furthermore, the majority of cases occurred in school children and the school outbreaks started the epidemic in the rest of the population. Cumulative age-specific incidence was not different from previous epidemic seasons except in the age group 15-64 years, although the weekly age-specific incidence rates were highest during the influenza season 2009/10.

The most significant features of this epidemic, besides the rapid establishment of community transmission, were geographic variability in the number of hospitalized patients, more severe and sustained capacity problems in intensive care units, an increased risk of severe illness in adults, and abrupt cessation of community transmission (16). Our hospitalization and mortality rates are compatible with the data from other countries (14,17-19). The hospitalization rates from 14-162 per 100 000 inhabitants in our province were higher than 2.0-31.8 per 100 000 observed in Southern hemisphere countries, while the mortality rates were similar (18,19). Additional hospital bed and staff capacities were used to provide medical care for patients with SARI at the peak of the outbreak in districts with the highest hospitalization rates. The difference in CFRs of hospitalized cases among districts was a result of different hospital admittance criteria. In North Bačka district, only the most severe cases were admitted, which reflected in the higher proportion of deaths among the admitted cases when CFR in hospitalized cases was calculated.

In the Netherlands, the hospitalization rates due to pandemic influenza by age group ranged from 2.8 to 62.7 (overall 13.1) per 100 000 and mortality rates ranged from 0.05 to 0.61 (overall 0.32) per 100 000 (8). Hospitalization and mortality rates in Vojvodina due to pandemic influenza by age group ranged more widely. The hospitalization rates were lowest in the oldest group of participants and highest in the age group 15-19 years, while the highest mortality rate was observed in the age group of 20-64 years. In Brazil, the most frequently hospitalized were younger age groups and they had the highest mortality rates (20). As opposed to this, in New South Wales and the Netherlands the highest age-specific hospitalization rates and mortality rates were found in older age groups (10,16).

The presence of co-morbidity represented a greater risk factor of death in our population. Delay in hospital treatment commencement increased the severity of the illness and influenced its outcome, as it was shown in other countries (21,22).

During the outbreak, we confirmed 46% of sampled patients with SARI and 75% of sampled sentinel ILI cases. Higher percentage of negative results of polymerase chain reaction in patients with SARI can be explained by the fact that antiviral treatment was applied immediately after the hospital admission.

Sentinel surveillance and high quality data of hospitalized cases were essential for health service planning and responding during the pandemic H1N1 2009 influenza (23). In Osaka, Japan, in May 2009 school closure slowed down the outbreak (24). Our results confirmed such results, suggesting that school closure most likely contributed to the influenza A(H1N1)v transmission control. School closure is more effective when attack rates in children are higher than in adults (25), which was the case in Vojvodina. Besides, the sentinel surveillance showed that school closure had an effect on the intensity of epidemic spreading, while SARI surveillance showed that the measure reduced the number of hospital admissions and the pressure on hospitals.

School closure has not always had a mitigating effect on pandemic influenza, most probably due to its duration and delay in implementation (25). When schools in Vojvodina were closed for one week, influenza epidemic was slowed down and the intensity of clinical activity decreased from a very high to high level but the outbreak was not stopped. There was an epidemiological recommendation to extend the period of closure, so that when schools were closed for the second time for three weeks, the intensity of clinical activity decreased to a low level. It is still unclear whether it was the second school closure that ended the outbreak or it ended naturally.

In order to control influenza transmission with vaccines, high vaccine coverage is needed in high risk groups, as well as in the general population. Vaccination gives the best results if vaccines are given before the beginning of the outbreak (26). Vaccination in Vojvodina started at the end of the outbreak, with low coverage and could not influence the course of the epidemic.

Sentinel ILI and SARI surveillance, both followed by virological surveillance, seem to be the optimal methods to monitor the full scope of the influenza pandemic (from mild to severe influenza) in Vojvodina. This approach enabled us to propose timely and quality decisions to health authorities regarding the public health control measures.

## References

[1] World Health Organization. Pandemic (H1N1) 2009-update 62 (revised 21 August 2009). Available from: http://www.who.int/csr/don/2009_08_21/en/index.html*.* Accessed: March 1, 2011.

[2] World Health Organization. Interim WHO guidance for the surveillance of human infection with swine influenza A(H1N1) virus 27 April 2009. Available from: http://www.who.int/csr/disease/swineflu/WHO_case_definitions.pdf*.* Accessed: March 1, 2011.

[3] Loncarevic G, Payne L, Kon P, Petrovic V, Dimitrijevic D, Knezevic T (2009). Public health preparedness for two mass gathering events in the context of pandemic influenza (H1N1) 2009 – Serbia, July 2009. Euro Surveill.

[4] European Center for Disease Control and Prevention. CDC. Overview of surveillance of influenza 2009/2010 in EU/EEA. Technical document Available from: http://ecdc.europa.eu/en/publications/Publications/0909_TED_Overview_of_Surveillance_of_Influenza_2009-2010_in_EU-EEA.pdf Accessed: March 1, 2011.

[5] Aguilera JF, Paget WJ, Mosnier A, Heijnen ML, Uphoff H, van der Velden J (2003). Heterogeneous case definitions used for the surveillance of influenza in Europe.. Eur J Epidemiol.

[6] Institute for Health Protection of the Republic of Slovenia. Influenza-like illness and acute respiratory infections in the season1999/2000 [in Slovenian]. Available from: http://www.ivz.si/?ni=165&pi=5&_5_Filename=1659.pdf&_5_MediaId=1659&_5_AutoResize=false&pl=165-5.3*.* Accessed: March 9, 2011.

[7] Ministry of Health of the Republic of Serbia. New guidelines for A(H1N1)v influenza surveillance in the Republic of Serbia. Available from: http://www.minzdravlja.info/downloads/2009/Avgust/Avgust%202009%20Nove%20Smernice%20AH1N1.pdf *Created 04.08.2009.* Accessed: March 9, 2011.

[8] World Health Organization. CDC realtime RTPCR (rRTPCR) Protocol for Detection and Characterization of Swine Influenza (version 2009). Available from: http://www.who.int/csr/resources/publications/swineflu/CDCrealtimeRTPCRprotocol_20090428.pdf Accessed: March 1, 2011.

[9] World Health Organization. Strategic Advisory Group of Experts on Immunization – report of the extraordinary meeting on the influenza A (H1N1) 2009 pandemic, 7 July 2009. Weekly Epidemiological Record, No. 30, 24 July 2009. Available from: http://www.who.int/wer/2009/wer8430.pdf Accessed: March 1, 2011. 19630186

[10] van 't Klooster TM, Wielders CC, Donker T, Isken L, Meijer A, van den Wijngaard CC (2010). Surveillance of hospitalisations for 2009 pandemic influenza A(H1N1) in the Netherlands, 5 June - 31 December 2009. Euro Surveill.

[11] Carrat F, Tachet A, Rouzioux C, Housset B, Valleron AJ (1999). Evaluation of clinical case definitions of influenza: detailed investigation of patients during the 1995-1996 epidemic in France.. Clin Infect Dis.

[12] Centers for Disease Control and Prevention (2009). Surveillance for pediatric deaths associated with 2009 pandemic influenza A (H1N1) virus infection - United States, April-August 2009.. MMWR Morb Mortal Wkly Rep.

[13] Institut de veille sanitaire (InVS). Influenza A(H1N1) 2009 epidemiological report, situation as of 21 July 2009 [in French]. Bulletin grippe A (H1N1) 2009 [serial on the Internet]. Available from: http://www.invs.sante.fr/surveillance/grippe_dossier/points_h1n1/grippe_A_h1n1_220709/Bulletin_grippe_22_07_09.pdf*.* Accessed: March 1, 2011.

[14] Baker MG Wilson N Huang QS Paine S Lopez L Bandaranayake D Euro Surveill 2009 14 pii=19319 Pandemic influenza A(H1N1)v in New Zealand: the experience from April to August 2009Euro Surveill. 2009;14.pii: 19319 19712648 10.2807/ese.14.34.19319-en

[15] Dawood FS, Jain S, Finelli L, Shaw MW, Lindstrom S, Novel Swine-Origin Influenza A (H1N1) Virus Investigation Team (2009). Emergence of a novel swine-origin influenza A (H1N1) virus in humans.. N Engl J Med.

[16] New South Wales public health network (2009). Progression and impact of the first winter wave of the 2009 pandemic H1N1 influenza in New South Wales, Australia. Euro Surveill.

[17] Cullen G, Martin J, O'Donnell J, Boland M, Canny M, Keane E (2009). Surveillance of the first 205 confirmed hospitalised cases of pandemic H1N1 influenza in Ireland, 28 April - 3 October 2009. Euro Surveill.

[18] Baker M, Kelly H, Wilson N. (2009). Pandemic H1N1 influenza lessons from the southern hemisphere. Euro Surveill.

[19] State Population Health Emergency Operations Centre. Queensland Government. Pandemic (H1N1) 2009. Influenza in Queensland 2009 Weekly Report for Week 41. 12 October 2009. Registered document no. CH003112. Available from: http://www.docstoc.com/docs/16058979/Influenza-in-Queensland-2009-Weekly-Report-12-October-2009*.* Accessed: March 1, 2011.

[20] Oliveira W, Carmo E, Penna G, Kuchenbecker R, Santos H, Araujo W (2009). Pandemic H1N1 influenza in Brazil: analysis of the first 34,506 notified cases of influenza-like illness with severe acute respiratory infection (SARI). Euro Surveill.

[21] Argentina Ministry of Health. Pandemic (H1N1) 2009: Weekly epidemiological report no 34. 4 September 2009. [in Spanish]. Available from: http://www.msal.gov.ar/archivos/Informe_SE_34-_ARG_COM.pdf*.* Accessed: March 1, 2011.

[22] Mayoral Cortes JM, Puell Gomez L, Perez Morilla E, Gallardo Garcia V, Duran Pla E, Fernandez Merino JC (2009). Behaviour of the pandemic H1N1 influenza virus in Andalusia, Spain, at the onset of the 2009-10 season. Euro Surveill.

[23] Martin J, O'Donnell J, Igoe D, O'Hora A, Thornton L, Murphy N (2009). Enhanced surveillance of initial cases of pandemic H1N1 2009 influenza in Ireland, April-July 2009. Euro Surveill.

[24] Nishiura H, Castillo-Chavez C, Safan M, Chowell G. (2009). Transmission potential of the new influenza A(H1N1) virus and its age-specificity in Japan. Euro Surveill.

[25] Sypsa V, Hatzakis A. (2009). School closure is currently the main strategy to mitigate influenza A(H1N1)v: a modeling study. Euro Surveill.

[26] Centers for Disease Control and Prevention. Prevention and control of influenza with vaccines: recommendations of the Advisory Committee on Immunization Practices [ACIP], 2010. MMWR 2010;59[No. RR-8]. Available from: http://www.cdc.gov/mmwr/preview/mmwrhtml/rr5908a1.htm*.* Accessed: March 9, 2011. 20689501

